# Fostering interprofessional communication through case discussions and simulated ward rounds in nursing and medical education: A pilot project

**DOI:** 10.3205/zma001027

**Published:** 2016-04-29

**Authors:** Birgit Wershofen, Nicole Heitzmann, Esther Beltermann, Martin R. Fischer

**Affiliations:** 1 Klinikum der Universität München, Institut für Didaktik und Ausbildungsforschung in der Medizin, München, Germany

**Keywords:** Interprofessional education, interprofessional communication, case discussion, simulated ward rounds

## Abstract

**Background: **Poor communication between physicians and nursing staff could result in inadequate interprofessional collaboration with negative effects on patient health. In order to ensure optimal health care for patients, it is important to strengthen interprofessional communication and collaboration between physicians and nurses during their education.

**Aim: **The aim of this project is to foster communication for medical and nursing students through interprofessional case discussions and simulated ward rounds as a form of training.

**Method:** In 2013-15 a total of 39 nursing students and 22 medical students participated in eight seminars, each covering case discussions and simulated ward rounds. The seminar was evaluated based on student assessment of the educational objectives.

**Results: **Students who voluntarily signed up for the seminar profited from the interprofessional interaction and gathered positive experiences working in a team.

**Conclusion: **Through practicing case discussions and ward rounds as a group, interprofessional communication could be fostered between medical and nursing students. Students took advantage of the opportunity to ask those from other profession questions and realized that interprofessional interaction can lead to improved health care.

## Zusammenfassung

**Hintergrund: **Kommunikationsdefizite zwischen Ärztinnen^1^ und Pflegepersonen können eine schlechte interprofessionelle Zusammenarbeit bewirken, die zu negativen gesundheitlichen Auswirkungen für Patienten führen können. Eine Förderung der interprofessionellen Kommunikation und Zusammenarbeit ist bereits in der Ausbildung von Ärztinnen und Pflegepersonen wichtig um zu einer optimalen Patientenversorgung beizutragen.

**Zielsetzung: **Ziel des vorliegenden Unterrichtsprojekts ist die Förderung der interprofessionellen Kommunikation für Gesundheits- und Krankenpflegeschülerinnen und Medizinstudierende durch gemeinsame Fallbesprechungen und Visitensimulationstrainings.

**Methodik:** Im Zeitraum von 2013 - 2015 nahmen insgesamt 39 Gesundheits- und Krankenpflegeschülerinnen und 22 Medizinstudierende an acht Seminaren teil, die jeweils Fallbesprechungen und Visitensimulationstrainings beinhalteten. Die Evaluation der Seminare erfolgte über die Einschätzung der Lernenden nach Lernzielen.

**Ergebnisse: **Teilnehmerinnen, die sich freiwillig zu den Seminaren anmeldeten, profitierten durch den interprofessionellen Austausch und machten positive Erfahrungen über das Arbeiten im Team.

**Schlussfolgerungen:** Durch die Übungen der gemeinsamen Fallbesprechungen und Visitensimulationen konnte die interprofessionelle Kommunikation bei Gesundheits- und Krankenpflegeschülerinnen und Medizinstudierenden gefördert werden. Die Lernenden nutzten die Möglichkeit bei der anderen Profession nachzufragen und machten die Erfahrung, dass ein interprofessioneller Austausch zu einer verbesserten Patientenversorgung beitragen kann.

## Introduction

In the cooperation between physicians and nursing professionals a communication deficit is prevailing. This becomes obvious by the fact that professional opinions about patients are insufficiently expressed and shared. Moreover, the other professional’s potential for action is frequently underestimated and remain unknown to the other [1]. This communication deficit can result in poor collaboration and negatively impact patients [2]. Hence, preparing students during their education for future collaboration seems reasonable [3], [4], [5] as a way to contribute to efficient, cost-effective and high-quality health care [6], [7], [http://www.aacn.nche.edu/education-resources/ipecreport.pdf cited 15-08-2015]. Until today, interprofessional communication training has neither been widely offered nor investigated in undergraduate programs [8]. Within the current project, a teaching strategy was developed to promote communication between medical and nursing students. Emphasis was placed on becoming familiar with the perspectives and decision-making possibilities of the respectively other profession, rather than on practicing clear communication in critical situations (e.g. SBAR) [9], [10].

When developing the teaching concept, the contact hypothesis [11], [12] was applied as the theoretical framework. The basic aspect of the contact hypothesis is the communication between two groups who exchange knowledge and share their perspectives during exercises. In order to achieve a deconstruction of stereotypes and a positive change in attitudes during the exercises, a cooperative atmosphere, a shared goal, equal status among the groups and the guidance of instructors are essential [11]. This seems appropriate if interprofessional learning takes place in interaction between students from two or more professions [13]. As a result, improved interaction can occur and a more open communication can be fostered between the professional groups [3], [14], [15], [16], [17], [18].

For this project, two situations were selected that commonly occur in clinical practice, and for which interprofessional communication is crucial: interprofessional case discussions and ward rounds.

Interprofessional case discussions are an important teaching method to present complex problems [2], [3], [7]. Case discussions encompass a communicative process and represent a problematic patient situation: to achieve patient-centered solutions, ideas are discussed within the team [19], [20], [21]. Through this exchange, students have the opportunity to better understand the point of view of the other profession. In addition it has been shown that looking at problem from a different perspective, such as the perspective from another health care profession, leads to more effective, safer health care [16] and is more closely to patient-centered care [3], [13]. In the current study, specific frameworks were used to structure the case discussions [7]. In clinical practice case discussions frameworks for addressing ethical questions are well-established [21], [22], [23]. However, a framework specifically for general clinical healthcare situations is almost unknown [7]. In this project, the case discussion frameworks are used as a tool to learn how to conduct case discussions. The case discussions are meant to achieve a coordinated plan for therapy. After discussing the clinical case within the team, the students present the treatment plan to the patient in a simulated ward round. This step, moreover, makes it possible to practice communication with patients as well. 

The aim of this teaching project is to foster interprofessional communication for nursing staff and physicians through joint case discussions and simulated ward rounds during initial education. The question arising from this is whether or not the interprofessional communication practiced by the students will be subjectively improved as a result of attending the interprofessional seminar.

The teaching concept was first realized as a pilot project in preparation for implementation in the nursing and medical curricula. The Robert Bosch Stiftung provided support for the pilot project within the scope of its grant program, “Operation Team – Interprofessional Learning in the Health Care Professions”.

## Project description

**Figure 1:**
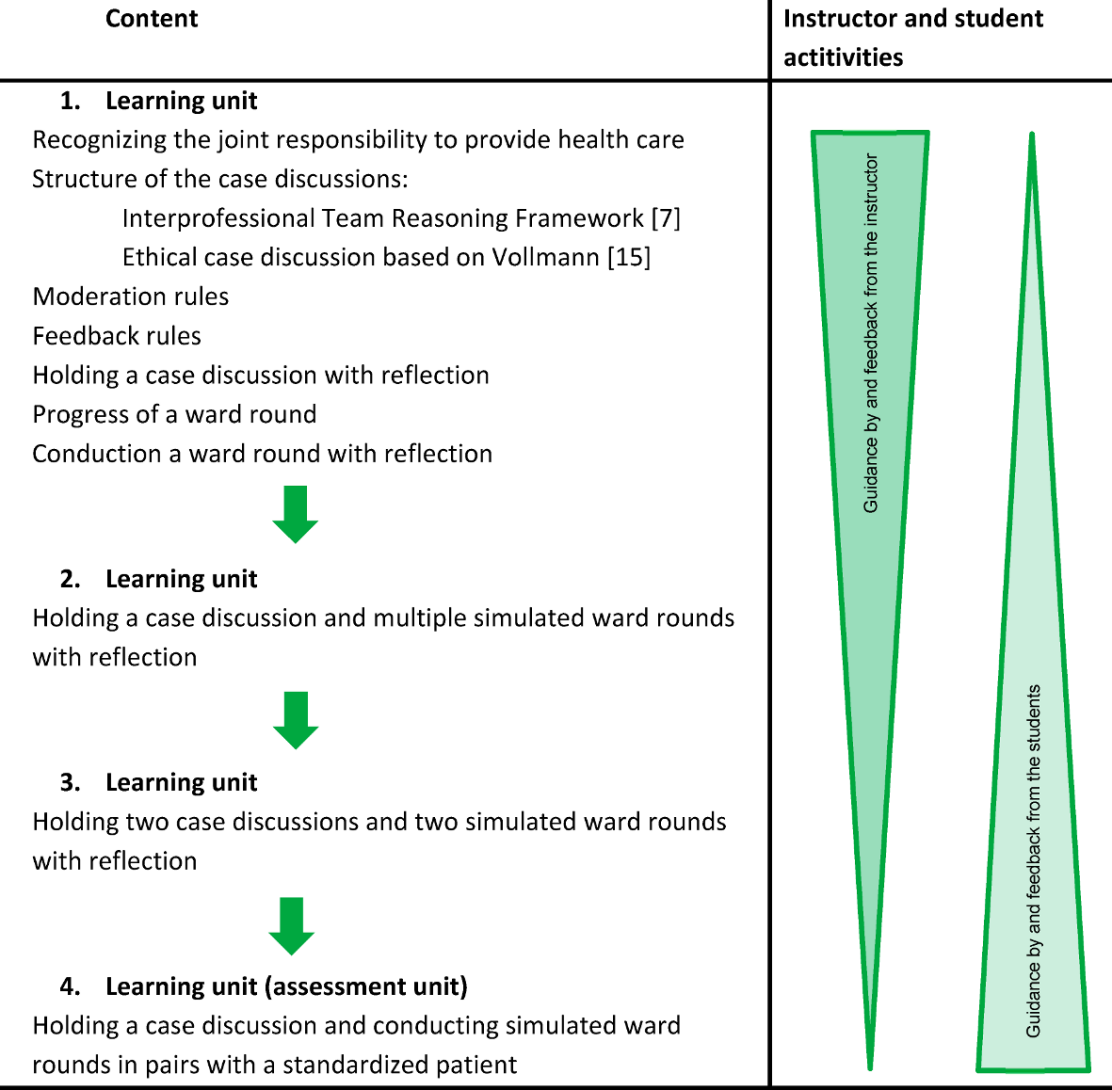
Overview of the seminar units

The pilot project was designed as a seminar and covered four instructional units each consisting of four lesson units. Figure 1 (Fig. 1) gives an overview of the seminar. At the beginning of the first lesson, the different and common tasks in health care were covered. This introduction was meant to give insight into the responsibilities of the respective other profession and to convey to the students, that both professions share the common goal of providing health care. 

Moreover, the exchange that takes place between the students is an important aspect of the contact hypothesis [11]. The students are introduced to two models for case discussions: the Interprofessional Team Reasoning Framework [7] and case discussion based on Vollmann [21]. Afterwards, rules for moderating and giving feedback are introduced. The first case discussion is moderated by a teacher. Students take on the roles in the case discussions. Following the progress of the case discussion and the result are reflected, including considerations on alternatives. Next, students are instructed on how to conduct a joint ward round. The instructor demonstrates how to communicate the results of the case discussion to the patients in context of a ward round. When doing this, a student assumes the role of patient. Finally, the reflection is guided by instructors and takes place with structured feedback on the simulations to augment the learning process [24].

During each of the second and third lesson units, one to two example cases are presented and used for the case discussion and ward round. The moderations of the case discussions and each of the related roles are conducted in alternation by the students. Over the course of the seminar, students increasingly take responsibility in leading the case discussion, simulated ward round, and the reflective exercises. The instructor’s responsibility is, based on the contact hypothesis [11], to provide support as needed. To encourage participants to see things from another perspective, students are offered to take on the role from the other profession.

There is a formative assessment at the end of the lesson unit, in which the group independently carries out a case discussion and then reflects upon it. The results of the case discussion are communicated by two students to a standardized patient in a simulated ward round. Every student is involved in a simulated scenario. The reflective process is supplemented by the feedback given by the standardized patient. At the end of the seminar, the participants receive a certificate documenting their attendance.

An important aspect of planning the seminar was selecting the instructors. The expertise in teaching is a crucial factor in enabling constructive interprofessional learning [25]. This is why the seminar was taught by physicians and nurses with teaching experience. The instructors were prepared for the interprofessional teaching situation in a joint meeting beforehand, which comprises the seminar content, knowledge about cooperative learning [26], attitudes and assumptions about stereotypes [17], [27], and the development of a professional identity [27]. Furthermore, the conditions and responsibilities arising from the theoretical framework of the contact hypothesis [11] were discussed, for instance how to create a cooperative learning atmosphere.

Case-based learning is a frequently used approach to initiate the learning process for students in the health professions [28]. For this reason, the development of cases for this seminar was another central aspect. The cases are based on real challenges in providing patient care. The heuristic matrix of Darmann-Finck [29] was used as a focusing element and to review the cases for educational content. The matrix covers the points of view held by nurses, patients and their relatives, and the institution and healthcare system in light of technical, practical and emancipatory cognitive interests. In addition, the physician’s perspective was also included.

A comprehensive knowledge is needed to grasp the complex problems in the presented cases. Therefore the seminar was offered to nursing students in their third year of study and medical students either in their ninth semester or fifth year (practical phase) of study. Different group constellations were tested: interprofessional groups, consisting of nursing and medical students and uniprofessional groups.

## Methods

Many questionairs have been developed in the English-speaking countries to evaluate interprofessional education [30], [31], [32]. The extent to which German translations of these international scales can be used due to differences in educational systems has not been ascertained. For the seminar, a questionnaire was developed based on evaluation surveys used internally by the medical school at the Ludwig Maximilian University (LMU) in Munich, Germany.

The questionnaire contained 18 items having a six-point Likert scale (1=completely true to 6=completely false) and two qualitative questions that referred to positive aspects and suggestions for improvement. A total of 61 students attended the seminar. The participants were enrolled at LMU and two nursing schools in Munich - Berufsfachschule für Krankenpflege Maria Regina (groups 1-7) and Private Berufsfachschule für Krankenpflege Dritter Orden und Barmherzige Brüder (group 8). Table 1 (Tab. 1) gives an overview of the participant numbers and distribution according to profession and sex.

The nursing students in the first three groups all came from one class. To facilitate organization of the practical components, the seminar was held during regular class time during the first year of piloting. Groups 1 and 2 worked interprofessionally, while group 3 (consisting only of nursing students) served as a control. The seminar was held in parallel for groups 1, 2 and 3. Over the course of the seminar, the nursing students complained about the necessity to attending. Because of their participation on a not voluntary basis, their data were excluded from the analysis. A further consequence was that the subsequent seminars were held outside of regular class time.

Participation for the medical students was voluntary. Despite great efforts to recruit with campaigns, emails, announcements in instructors and recommendations only a small number of medical students signed up. The interprofessional groups 1 and 2 each had only one medical student as a result. The control group (group 4) consisted of four medical students. Further 15 medical students (groups 5-8) were then recruited in an extensive effort to encourage participation.

## Results

In the seminars students had the opportunity to practice structured communication in the form of a case discussion and to present the results in simulated ward rounds. The intended comparison of the groups was not possible due to the low number of medical students in the first groups and the change from regular class time to voluntary participation for the nursing students. As reported, only the data of persons willingly participating in the seminar were analyzed. The results refer to the nursing students in groups 5-8 and medical students in groups 1, 2, and 4-8. The low number of participants limits the statistical analysis and therefore, only descriptive data are reported. Nevertheless, the data provide valuable information for continuation and implementation in the medical and nursing curricula. The data analysis was carried out with SPSS (version 22).

Overall, 12 female and two male nursing students participated, of which one male student was unable to complete the seminar for work-related reasons. Of the 22 medical students, 13 were female and nine male. The response rate from the students who completed the seminar was 100%. The mean age of the nursing students was 22.5 years (range 20-29 years), that of the medical students was 28 years (range 21-45 years).

The most important student responses on the questionnaires are compiled in table 2 (Tab. 2) and table 3 (Tab. 3). 

It can be seen that the students felt they were able to lead interprofessional case discussions. 

Moreover, the students learned what they need to pay attention to interprofessional communication (*M*=1.66) and took advantage of the opportunity to ask questions to the other profession in order to understand their occupational perspectives (*M*=1.60). In addition, the students were open to the views of the other professional group (*M*=1.57). During the seminar students also realized that the arguments presented by the other professional group led to a change in their own actions and decision-making, something that can contribute to improved patient care (*M*=1.54).

Student assessment of acceptance is presented in table 3 (Tab. 3). The level of acceptance is determined with the three following questions: would the students recommend the seminar to others; were they interested in more interprofessional seminars; and if the cases were appropriate for their learning. It appears that the participants would recommend the seminar to their fellow students at the vocational schools or university (*M*=1.34). Also, the interest in taking other interprofessional seminars is very high (*M*=1.63). The cases were also evaluated as appropriate (*M*=1.86).

As a final question, students were asked to describe what they particularly liked about the seminar and what could have been better. In their responses, students described the insights into the other profession’s view as an added bonus. The opportunity to work together and share ideas on cases was mentioned as a very good strategy to prepare for practice. In addition, the positive learning atmosphere was valued. The structured teamwork was identified as being helpful. The students reported that the time for joint learning was sufficient to allow them to come together as a team. Furthermore, the feedback was experienced as being helpful. Suggestions for improvement included later starting times for the seminar and even more detailed information about the cases.

## Discussion

These results point out that the students felt an improvement in their ability to communicate interprofessionally. Some studies show in a similar manner that interactive exercises contribute to improving interprofessional communication [14], [17], [18]. In addition, the students were able to identify a change in their attitude that contributes to improving patient care, which is an important goal of interprofessional collaboration [8]. The evaluation of the seminar is an initial step toward determining the subjective achievement of the learning objectives and the acceptance of the seminar. Since suitable evaluation tools for interprofessional education are presently lacking in the German-speaking countries, a student evaluation questionnaire for the seminar was developed. In a second step this questionnaire is to serve as the basis for developing a standardized, validated evaluation tool. To gather detailed information, further evaluation methods (e.g. interviews, performance test) are planned for the next project phase.

The following discusses the challenges and changes for the pilot project and its results.

The application of the seminar schedule proved to be appropriate. The opportunity to change the perspective and take the other professions roles was not taken by all of the groups or students. The reason given for this by students was that they were sufficiently challenged with the structured case discussions, the demands of the cases, and with their own professional role.

Challenging and very time consuming was the recruitment of medical students. These campaigns were only partially successful. A personal recommendation of an advocate has been proven to be particularly beneficial in recruiting medical students. The great amount of 16 learning units hindered the participation in the seminar. Reducing the amount of time was not considered, because of students’ emphasis on the value to have time together and to work on the exercises.

The original intention to conduct the seminar during regular class time for nursing students was not perceived as a concession, but rather as an obligation. An important reason for the dissatisfaction of the nursing students in the first three groups was that their expectation of joint learning with an equal number of medical students was not met. This unfulfilled expectation contributed to the development of certain group dynamics that culminated in a rejection of the seminar. It became clear as a result that the selection of instructors with teaching experience is an important aspect, as well as the intensive preparation [13]. To date, there has been no teacher training concerning interprofessional teaching situations.

An important outcome of the pilot project is the necessity of voluntary participation in interprofessional seminars. Freeth et al. [13] emphasize the voluntary participation as an important motivational factor for students, which is confirmed in the realization the seminars as optional ones and is reflected in the student evaluations. Signing up voluntarily for an interprofessional communication seminar indicates a pre-existing curiosity and interest in the other profession.

To implement interprofessional communication as a permanent and essential part in education, the seminar should be included as an elective in the nursing and medical curricula. Regularly and repeatedly holding seminars underlines the importance of interprofessional communication for patient care for soon-to-be health care professionals. If further positive learning outcomes can be demonstrated, a next step should entail offering opportunities for interprofessional education to all students.

## Conclusion

Nursing and medical students experienced an improvement in interprofessional communication. This is a first step to improve interprofessional communication in day-to-day work, which could further lead to an improvement in patient care.

The seminar’s format and structure have proven to be successful. While conducting the seminar, positive learning effects were seen for nursing and medical students who participated voluntarily. The implementation of this seminar in the nursing and medical curricula is planned as a next step. Importance will be placed on having an equal number of students from each profession and on the voluntary nature of participation. Extensive recruiting campaigns at the medical schools and vocational schools will be initiated for this purpose. Expansion of the course offering to include other health professions is being considered.

A program to train the trainer is planned to ensure the quality in teaching interprofessional seminars.

From the instructors’ point of view it is important to understand the effects of interprofessional education in order to create positive learning situations. For instance, the extent to which improvement in interprofessional communication can be traced to the use of structured case discussions or simply to the opportunity to interact in a shared learning setting is still unknown. The question also arises whether changes in perspective or attitude are based on prior interest in the other professional group or are a result of experiencing teamwork. The presented project offers a starting point with the potential to address other issues involving interprofessional education.

## Funding

The project is received a grant form the Robert Bosch Stiftung (project number 32.5.1316.0004.0)

## Competing interests

The authors declare that they have no competing interests.

## Figures and Tables

**Table 1:**
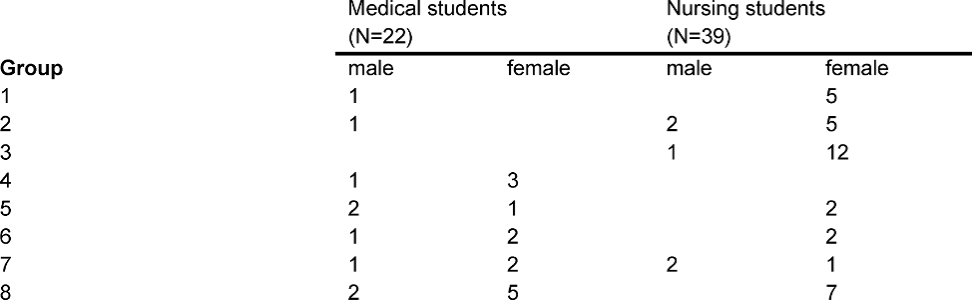
Group composition

**Table 2:**
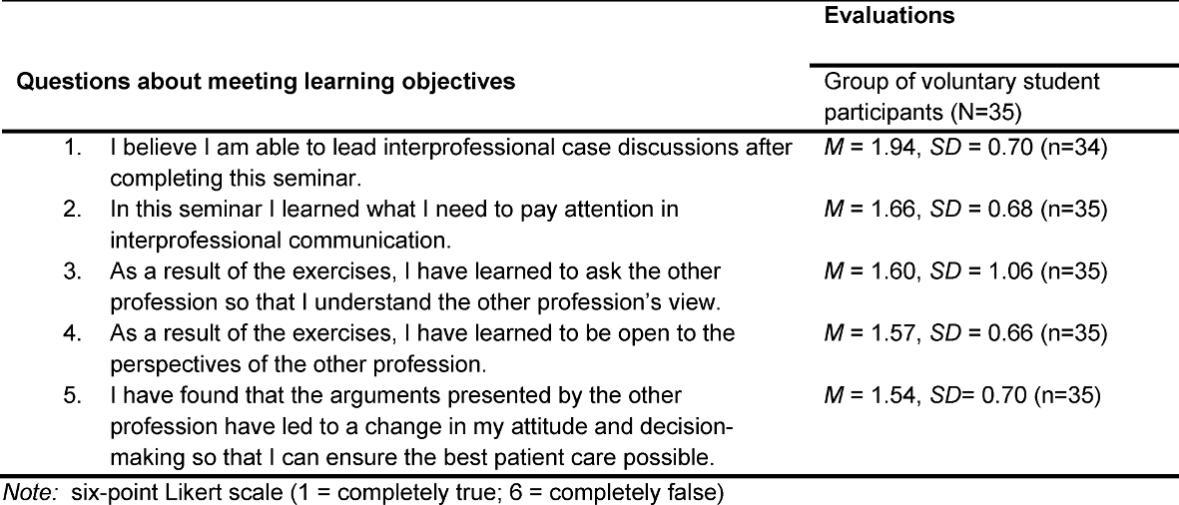
Student evaluation of the learning objectives

**Table 3:**
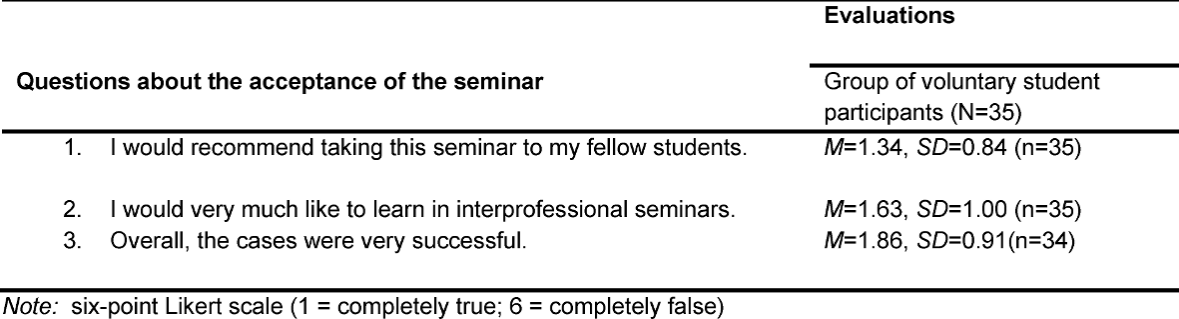
Student evaluation of acceptance

## References

[1] Sieger M, Ertl-Schmuck R, Bögemann-Großheim E (2010). Interprofessionelles Lernen als Voraussetzung für interprofessionelles Handeln – am Beispiel eines interprofessionell angelegten Bildungs- und Entwicklungsprojektes für Gesundheitsberufe. Pflege Gesellschaft.

[2] Zwarenstein M, Goldman J, Reeves S (2009). Interprofessional collaboration: effects of practice-based interventions on professional practice and healthcare outcomes. Cochrane Database Syst Rev.

[3] Bridges DR, Davidson RA, Odegard PS, Maki IV, Tomkowiak J (2011). Interprofessional collaboration: three best practice models of interprofessional education. Med Educ Online.

[4] Sachverständigenrat (2007). Gutachten 2007 des Sachverständigenrats zur Begutachtung der Entwicklung im Gesundheitswesen.

[5] Weltgesundheitsorganisation (2010). Framework for action on interprofessional education and collaborative practice.

[6] Robert Bosch Stiftung (2011). Memorandum, Kooperation der Gesundheitsberufe Qualität und Sicherstellung der zukünftigen Gesundheitsversorgung.

[7] Packard K, Chehal H, Maio A, Doll A, Furze J, Huggett K, Jensen G, Jorgensen D, Wilken M, Qi Y (2012). Interprofessional Team Reasoning Framework as a Tool for Case Study Analysis with Health Professions Students: A Randomized Study. J Res Interprof Pract Educ.

[8] Bachmann C, Abramovitch H, Barbu CG, Cavaco AF, Elorza RD, Haak R, Loureiro E, Ratajska A, Silverman J, Winterburn S, Rosenbaum M (2013). A European consensus on learning objectives for a core communication curriculum in health care professions. Patient Educ Counc.

[9] Manning ML (2006). Improving clinical communication through structured conversation. Nurs Econ.

[10] DeMeester K, M (2013). Verspuy M, Monsieurs KG, VanBogaert P. SBAR improves nurse–physician communication and reduces unexpected death: A pre and post intervention study. Resuscitation.

[11] Allport GW (1979). The nature of prejudice.

[12] Hewstone M, Brown R, Hewstone M, Brown R (1986). Contact is not enough: An intergroup perspective on the 'contact hypothesis'. Contact and conflict in intergroup encounters.

[13] Freeth D, Hammick M, Reeves S, Koppel I, Barr H (2005). Effective Interprofessional Education: Development, Delivery & Evaluation.

[14] Maeno T, Takayashiki A, Anme T, Tohno E, Maeno T, Hara A (2013). Japanese students' perception of their learning from an interprofessional education program: a qualitative study. Int J Med Educ.

[15] Manser T, Foster S (2011). Effective handover communication: an overview of research and improvement efforts. Best Pract Res Clin Anaesthesiol.

[16] Wakefield AB, Carlisle C, Hall AG, Attree MJ (2008). The expectations and experiences of blended learning approaches to patient safety education. Nurse Educ Pract.

[17] Carpenter C (1995). Doctors and nurses: stereotype and stereotype change in interprofessional education. J Interprof Care.

[18] Keller KB, Eggenberger TL, Belkowitz J, Sarsekeyeva M, Zito AR (2013). Implementing successful interprofessional communication opportunities in health care education: a qualitative analysis. Int J Med Educ.

[19] Binner U, Ortmann K, Zimmermann RB, Zirnstein J (2012). Die Organisation und Durchführung von Fallkonferenzen - ein Leitfaden.

[20] Steinkamp N, Gordijn B (2000). Die Nimwegener Methode für ethische Fallbesprechungen. Rhein Ärztebl.

[21] Vollmann J, Dörries A, Neitzke G, Simon A, Vollmann J (2010). Ethische Falldiskussionen. Klinische Ethikberatung.

[22] Marckmann G, Mayer F (2009). Ethische Fallbesprechungen in der Onkologie: Grundlagen einer prinzipienorientierten Falldiskussion. Onkologe.

[23] Gordijn B (2000). Ethische Diskussionen im Team. Schwester Pfleger.

[24] Fanning RM, Gaba DM (2007). The role of debriefing in simulation-based learning. Simul Healthcare.

[25] reeth D, Reeves S (2004). Learning to work together: using the presage, process, product (3P) model to highlight decisions and possibilities. J Interprof Care.

[26] Hammick M, Freeth D, Koppel I, Reeves S, Barr H (2007). A best evidence systematic review of interprofessional education. BEME Guide No. 9. Med Teach.

[27] Hind M, Norman I, Cooper S, Gill E, Hilton R, Judd P, Jones SC (2003). Interprofessional perceptions of health care students. J Interprof Care.

[28] Williams B, Brown T, McCook F, Boyle M, Palermo C, Molloy A, McKenna L, Scholes R, French J, McCall L (2011). A pilot study evaluating an interprofessional education workshop for undergraduate health care students. J Interprof Care.

[29] Darmann-Finck I, rtl-Schmuck R, Fichtmüller F (2010). Eckpunkte einer Interationistischen Pflegedidaktik. Theorien und Modelle der Pflegedidaktik.

[30] Parsell G, Bligh J (1999). The development of a questionnaire to assess the readiness of health care students for interprofessional learning (RIPLS). Med Educ.

[31] McFadyen A, Maclaren W, Webster V (2007). The Interdisciplinary Education Perception Scale (IEPS): an alternative remodeled sub-scale structure and its reliability. J Interprof Care.

[32] Heinemann GD, Schmitt MH, Farrell MP, Brallier SA (1999). Development of an attitudes toward health care team scale. Eval Health Prof.

